# Speed-dependent and mode-dependent modulations of spatiotemporal modules in human locomotion extracted via tensor decomposition

**DOI:** 10.1038/s41598-020-57513-w

**Published:** 2020-01-20

**Authors:** Ken Takiyama, Hikaru Yokoyama, Naotsugu Kaneko, Kimitaka Nakazawa

**Affiliations:** 1 Tokyo University of Agriculture and Technology, Department of Electrical Engineering and Computer Science, Nakacho, Koganei, Tokyo, Japan; 2 Rehabilitation Engineering Laboratory, Toronto Rehabilitation Institute, University Health Network, Toronto, Ontario Canada; 3 The University of Tokyo, Department of Life Sciences, Graduate School of Arts and Sciences, Tokyo, Japan

**Keywords:** Central pattern generators, Motor control

## Abstract

How the central nervous system (CNS) controls many joints and muscles is a fundamental question in motor neuroscience and related research areas. An attractive hypothesis is the module hypothesis: the CNS controls groups of joints or muscles (i.e., spatial modules) by providing time-varying motor commands (i.e., temporal modules) to the spatial modules rather than controlling each joint or muscle separately. Another fundamental question is how the CNS generates numerous repertoires of movement patterns. One hypothesis is that the CNS modulates the spatial and/or temporal modules depending on the required tasks. It is thus essential to quantify the spatial modules, the temporal modules, and the task-dependent modulation of these modules. Although previous attempts at such quantification have been made, they considered modulation either only in spatial modules or only in temporal modules. These limitations may be attributable to the constraints inherent to conventional methods for quantifying the spatial and temporal modules. Here, we demonstrate the effectiveness of tensor decomposition in quantifying the spatial modules, the temporal modules, and the task-dependent modulation of these modules without such limitations. We further demonstrate that tensor decomposition offers a new perspective on the task-dependent modulation of spatiotemporal modules: in switching from walking to running, the CNS modulates the peak timing in the temporal modules while recruiting more proximal muscles in the corresponding spatial modules.

## Introduction

How the central nervous system (CNS) controls the human body is a fundamental question. The CNS controls the body while somehow resolving a significant number of degrees of freedom (DoFs), in fact, more than is necessary to achieve the desired motions^1^. For example, during walking and running in daily life, large numbers of joints and muscles should be controlled in an orchestrated manner. The module hypothesis is an influential proposal regarding how such a tremendous number of DoFs can be managed^2–6^. According to this hypothesis, the CNS effectively reduces the number of DoFs to be managed by controlling groups of joints or muscles, referred to as spatial modules, rather than single joints or muscles separately. Accordingly, the time-varying motor commands sent to the spatial modules are referred to as temporal modules.

How the brain constructs various repertoires of motions is another fundamental question. One possible solution is that the brain modulates the spatiotemporal modules depending on the task at hand. On the one hand, temporal modules, rather than spatial modules, may be modulated for specific tasks, such as walking, running, executing various types of gaits, or responding to unpredictable perturbations while walking^7,8^. On the other hand, task-dependent modulation may be applied to spatial modules rather than to temporal modules for certain tasks, such as responding to unpredictable perturbations to maintain balance while standing^9^ and performing arm-reaching movements^10^, as well as in various types of movements of frogs^11^. According to our recent findings, a number of modules show task-dependent modulation during walking and running at various speeds^12^. In summary, there are several seemingly different perspectives on how the spatiotemporal modules are modulated depending on the task.

One possible reason why there is no agreed-upon perspective on the task-dependent modulations of the spatiotemporal modules is the limitations inherent to conventional methods. Conventional methods, such as principal component analysis (PCA)^13^ and non-negative matrix factorization (NNMF)^14^, are classified as matrix decomposition methods. Although matrix decomposition is suitable for investigating the dependence of joint angle and electromyographic (EMG) data on only two factors (i.e., spatial and temporal modules), there can be limitations when considering three or more factors, such as spatial modules, temporal modules, and the task-dependent modulations of those modules. For example, with matrix decomposition, the task-dependent modulation of a temporal module between two tasks has been discussed under the constraint of the same spatial module; similarly, the modulation of a spatial module has been discussed without considering the modulation of the corresponding temporal module. To overcome these constraints and enable the investigation of task-dependent modulations while extracting both spatial and temporal modules, methods for appropriately considering (more than) three factors can be suitable. In contrast to matrix decomposition, which is inherently suitable for analyzing two factors because of the dimensionality of matrix data (i.e., rows and columns), tensor decomposition has been proposed as a generalized version of matrix decomposition for analyzing (more than) three factors, consistent with the dimensionality of tensor data (i.e., rows, columns, and stacked matrices in the third dimension, in the case of a three-dimensional tensor)^15^. Tensor decomposition thus enables us to consider three factors simultaneously, i.e., we can investigate task-dependent modulations while extracting both spatial and temporal modules at the same time.

Here, we demonstrate the effectiveness of tensor decomposition in extracting spatial modules, temporal modules, and the task-dependent modulations of these spatiotemporal modules. Throughout this study, we rely on CANDECOMP/PARAFAC (CP) decomposition^15,16^ because it is a natural extension of conventional methods (i.e., PCA and NNMF). Concretely, CP decomposition extracts spatial and temporal modules in a similar manner to conventional methods; additionally, CP decomposition generates task-dependent modulations of these modules. Another important feature is that CP decomposition does not require orthogonality among the spatial modules, in contrast to PCA. Because the orthogonality in PCA is a matter of mathematical convenience rather than a requirement for the analysis of joint angles, CP decomposition can generate more plausible spatial modules than PCA can. Furthermore, CP decomposition is applicable to non-negative data (e.g., EMG data) with non-negativity constraints. For the reasons discussed above, we utilize CP decomposition, a tensor decomposition algorithm, to extract spatial modules, temporal modules, and the task-dependent modulations of these spatiotemporal modules.

A few previous studies have attempted to demonstrate the effectiveness of tensor decomposition in analyzing joint angle and EMG data^17–21^. In a previous study, tensor decomposition was applied to wrist EMG data, and the results demonstrated the task-dependent modulation of the spatiotemporal modules^17^. That study relied on a variant of Tucker decomposition, which is a more general type of tensor decomposition that subsumes CP decomposition. In Tucker decomposition, there are three free parameters: the number of spatial modules, the number of temporal modules, and the number of factors describing task-dependent modulations. Although this method offers high flexibility and generality, the determination of these three parameters requires considerable computational time. In general, several different combinations of parameters can provide the same performance in reconstructing the original data. It is thus difficult to determine the optimal parameters.

Other studies^18–21^ have relied on the application of matrix tri-factorization, a reduced version of Tucker decomposition (a detailed description is given in^17^), to EMG data to reveal the task-dependent modulation of individual spatiotemporal modules. The matrix tri-factorization algorithm is closely related to CP decomposition. Tri-factorization enables the estimation of spatial modules, temporal modules, and how those modules are recruited in each task. It has two free parameters: the number of spatial modules and the number of temporal modules. Again, several different combinations of parameters may provide the same performance in reconstructing the original data. Although it remains unclear how best to determine the optimal parameters, previous studies have proposed an *a posteriori* method of parameter determination using linear discriminant analysis (LDA)^18–21^. Although LDA worked well in those studies^18–21^, it generally requires certain assumptions about what information is inherent to either (both) the spatial or (and) temporal modules that are extracted (e.g., the direction in arm-reaching movements^20^). One advantage of tensor decomposition is its ability to enable the investigation of task-dependent modulation (i.e., a third factor) without any *a posteriori* analysis. CP decomposition is the simplest version of tensor decomposition and has only one free parameter to be determined (i.e., the number of combinations of spatial modules, temporal modules, and factors for describing the task-dependent modulations of those modules). Due to its simplicity, CP decomposition enables the investigation of task-dependent modulations without any *a posteriori* analysis while saving computational time. Because CP decomposition has not yet been applied to joint angle or EMG data, it may provide novel perspectives in task-dependent modulations of spatiotemporal modules in a more effective manner.

Here, we apply CP decomposition to joint angle and EMG data to investigate both speed-dependent and mode-dependent (walking vs. running) modulation, on which no consensus has yet been achieved, although various perspectives on the corresponding modulations have been presented. Joint angles and muscle activities exhibit a speed dependence, and this dependence differs for each joint angle and muscle^3,22^. These findings were reported without the extraction of any modules. One interesting result is that independent of speed, the three angles of the thigh, shank and foot are confined to a two-dimensional space^3^; similarly, there are only five basic EMG patterns associated with human locomotion^4^. These examples of dimensional reduction inherent in kinematic data and EMG data indicate the involvement of spatiotemporal modules in human locomotion. A later study suggested that the manner in which the dimensionality is reduced in kinematic data depends on the speed of movement^23^—specifically, fewer dimensions are sufficient to explain the original data at higher speeds—and that this tendency is diminished in Parkinson’s disease. Thus, it can be inferred that fewer spatiotemporal modules may be recruited at higher speeds than at lower speeds. In contrast, our previous study indicated that the number of spatiotemporal modules evident in EMG data increases with an increase in speed^12^. Other studies have reported that only temporal modules, rather than spatial modules, may be modulated depending on the mode of movement^4,24^. In particular, the peaks in some temporal modules can exhibit mode-dependent temporal shifts^24^. In summary, no consensus has yet been reached regarding the speed-dependent and mode-dependent modulation of spatiotemporal modules in human locomotion. In this study, we apply CP decomposition to joint angle and EMG data collected during walking and running at various speeds to elucidate how the spatiotemporal modules are modulated depending on both speed and mode.

We demonstrate that tensor decomposition enables us to clarify the task-dependent modulations of spatiotemporal modules inherent in joint angle and EMG data; in particular, we demonstrate the effective analysis of human walking and running at various speeds. From the joint angle data, we extract two types of modules: (1) modules that show increasing recruitment with increasing speed and (2) modules that are recruited mainly during running. Based on the EMG data, we demonstrate three types of modules: (1) modules that show increasing recruitment with increasing speed, 2) modules that are recruited mainly during walking, and (3) modules that are recruited mainly during running. By comparing the second and third types of modules evident in EMG data, we present a new perspective on how the recruitment of spatiotemporal modules depends on the movement mode (walking or running): the CNS switches between walking and running not only by modulating the temporal modules, as reported in previous studies^4,24^, but also by recruiting spatial modules involving more proximal muscles during running compared with walking.

## Results

Our program code can be downloaded from the website of the corresponding author.

### Tensor decomposition

**Figure 1:**
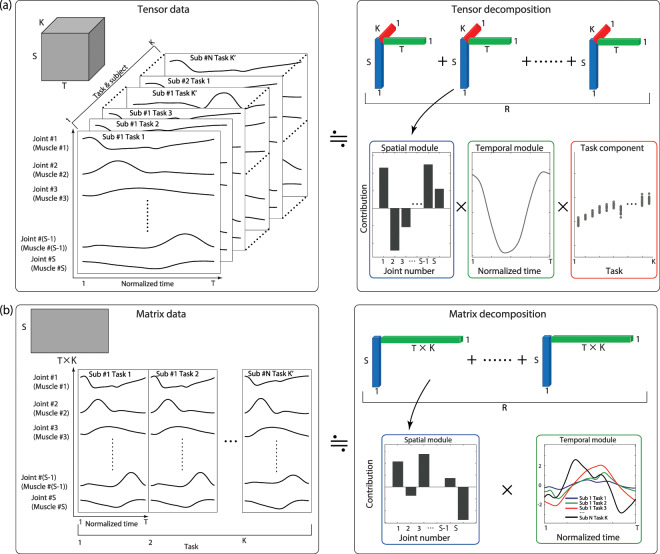
The concepts of tensor and matrix decomposition. (**a**) Tensor decomposition and CP decomposition, our focus in the current study. For decomposition, we construct a 3-dimensional array of data consisting of *S* columns, *T* rows, and *K* slices. After decomposition, we obtain spatial modules, as shown by the bar graph in the blue frame; temporal modules, as shown by the line plot in the green frame; and the task-dependent modulations of these modules, shown as circular dots in the red frame. (**b**) Matrix decomposition. To analyze *K* task datasets simultaneously, we need to establish an *S* × (*T* × *K*) matrix. After applying matrix decomposition, we obtain the spatial modules and the task-dependent modulations of the temporal modules.

**Figure 2:**
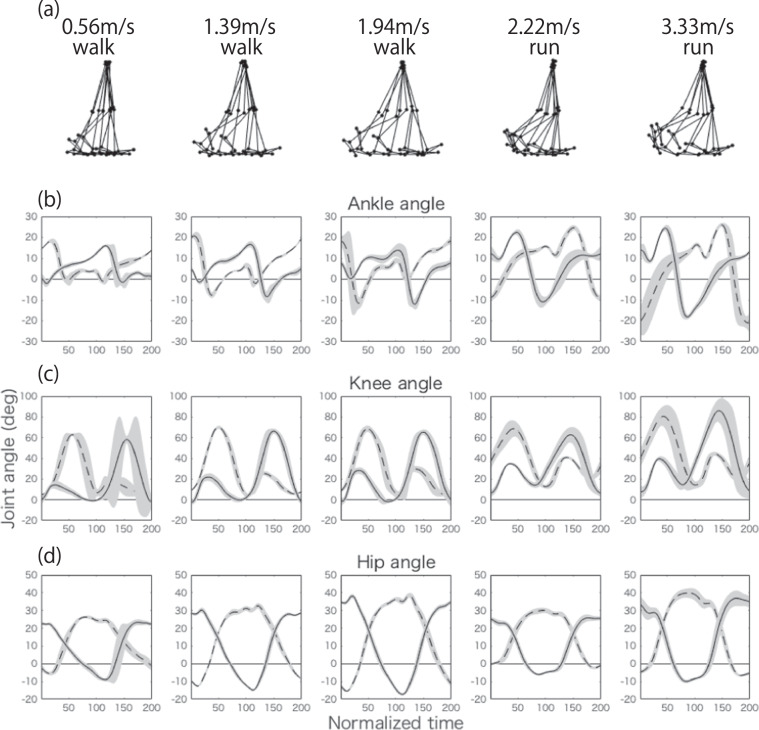
Joint angles of a representative subject at five representative speeds (0.56, 1.39, 1.94, 2.22, and 3.33 m/s). (**a**) Positions of the right hip, knee, and ankle every 20 time frames, normalized to their positions in the 200th time frame. At time 1, the right foot took off from the ground, and it returned at time 200. (**b–d**) Raw joint angle data for the ankles (panel (b)), knees (panel (c)), and hips (panel (d)) at each speed. The dotted lines indicate the angles for the left leg, and the solid lines indicate the angles for the right leg. The lines represent the joint angles averaged across 27 cycles, and the shaded areas represent the standard deviations of these angles. For tensor and matrix decomposition, we focus on the average joint angles after appropriate standardizations (see the Methods section for details). Table 1 summarizes the meanings of the positive and negative values for each joint.

We apply tensor decomposition to investigate the task-dependent modulation of the spatiotemporal modules extracted from joint angle and EMG data. The differences between tensor decomposition and matrix decomposition lie in how one prepares the original data for analysis and the obtained results. For tensor decomposition, the original data considered in the current study are arranged in 3-dimensional arrays. Notably, tensor decomposition can also be applied to arrays with more than 3 dimensions. Each array consists of a joint or muscle sequence (*S* columns in Fig. 1a), a temporal series (*T* rows in Fig. 1a), and a task sequence (*K* slices of *S* × *T* matrices in Fig. 1a). Throughout this paper, the word “task” broadly refers to motion under all types of conditions (i.e., walking or running at different speeds as shown in Fig. 2) for all subjects. Tensor decomposition enables the extraction of not only spatial and temporal modules but also the task-dependent modulations of these modules (Fig. 1a). Throughout this study, we use bar graphs to represent spatial modules, line plots to represent temporal modules, and circular dots to indicate task-dependent modulations, as shown in Fig. 1, following a previous study^16^.

We focus on CP decomposition throughout this paper. In CP decomposition, the (*i*, *j*, *k*)th element of the data tensor ***X*** ∈ **R**^*S*×*T*×*K*^, *X*_*i*,*j*,*k*_, is approximated as

1 $${X}_{i,j,k}\simeq \mathop{\sum }\limits_{r=1}^{R}\,{\lambda }_{r}{w}_{i,r}{p}_{j,r}{t}_{k,r}$$

where *S*, *T*, and *K* denote the number of joint angles or muscles, the number of time frames, and the number of tasks, respectively; *R* is the number of modules, to be determined *a priori*; *w*_*i*,*r*_ denotes the *i* th element of the *r* th spatial module, ***w***_*r*_ ∈ ***R***^*S*×1^; *p*_*j*,*r*_ denotes the *j* th element of the *r* th temporal module, ***p***_*r*_ ∈ **R**^*T*×1^; *t*_*k*,*r*_ denotes the *k* th element of the task-dependent modulation of the *r* th spatiotemporal module, ***t***_*r*_ ∈ **R**^*K*×1^ (Fig. 1a); and *λ*_*r*_ ≥ 0 denotes the scaling factor for the combination of the *r* th spatial module, the *r* th temporal module, and the task-dependent modulation of the *r* th spatiotemporal module under the conditions ${{\boldsymbol{w}}}_{r}^{T}{{\boldsymbol{w}}}_{r}=1$, ${{\boldsymbol{p}}}_{r}^{T}{{\boldsymbol{p}}}_{r}=1$, and ${{\boldsymbol{t}}}_{r}^{T}{{\boldsymbol{t}}}_{r}=1$. *λ*_*r*_ represents the contribution of the *r* th combination to explaining the original tensor data. A spatial module represents a group of joints or muscles to be controlled synchronously, and the associated temporal module represents the time-varying signal sent to this spatial module. The associated task-dependent modulation indicates the extent to which the corresponding spatiotemporal module is recruited in each task. This concept is closely related to dynamic motor primitives: time-varying motor commands are sent to muscles, the magnitude of such a motor command can change depending on task at hand, and the temporal width of such a motor command can change via the systems responsible for controlling movement time^25^.

The data corresponding to the *k* th task *X*_:,:,k_∈ **R**^*S×T*^ can be approximated as

2 $${X}_{:,:,k}\simeq \mathop{\sum }\limits_{r=1}^{R}\,{\lambda }_{r}{{\boldsymbol{w}}}_{r}{{\boldsymbol{p}}}_{r}^{T}{t}_{k,r},$$

which indicates that the spatiotemporal modules are independent of *k*, meaning that they are common across all tasks, and that the recruitment patterns of these modules are modulated depending on *t*_*k*,*r*_. The spatial modules, temporal modules, and task-dependent modulations are estimated so as to minimize the squared error between the original tensor data and the decomposed data:

3 $$E=\frac{1}{2}\sum _{i,j,k}\,{({X}_{i,j,k}-\mathop{\sum }\limits_{r=1}^{R}{\lambda }_{r}{w}_{i,r}{p}_{j,r}{t}_{k,r})}^{2},$$

with some constraints on *λ*_*r*_(≥0), *w*_*i*,*r*_, *p*_*j*,*r*_, and *t*_*k*,*r*_. For *w*_*i*,*r*_, *p*_*j*,*r*_, and *t*_*k*,*r*_, there are no constraints for the analysis of the joint angle data, and there are non-negativity constraints for the analysis of the EMG data (i.e., *w*_*i*,*r*_ ≥ 0, *p*_*j*,*r*_ ≥ 0, and *t*_*k*,*r*_ ≥ 0).

**Figure 3:**
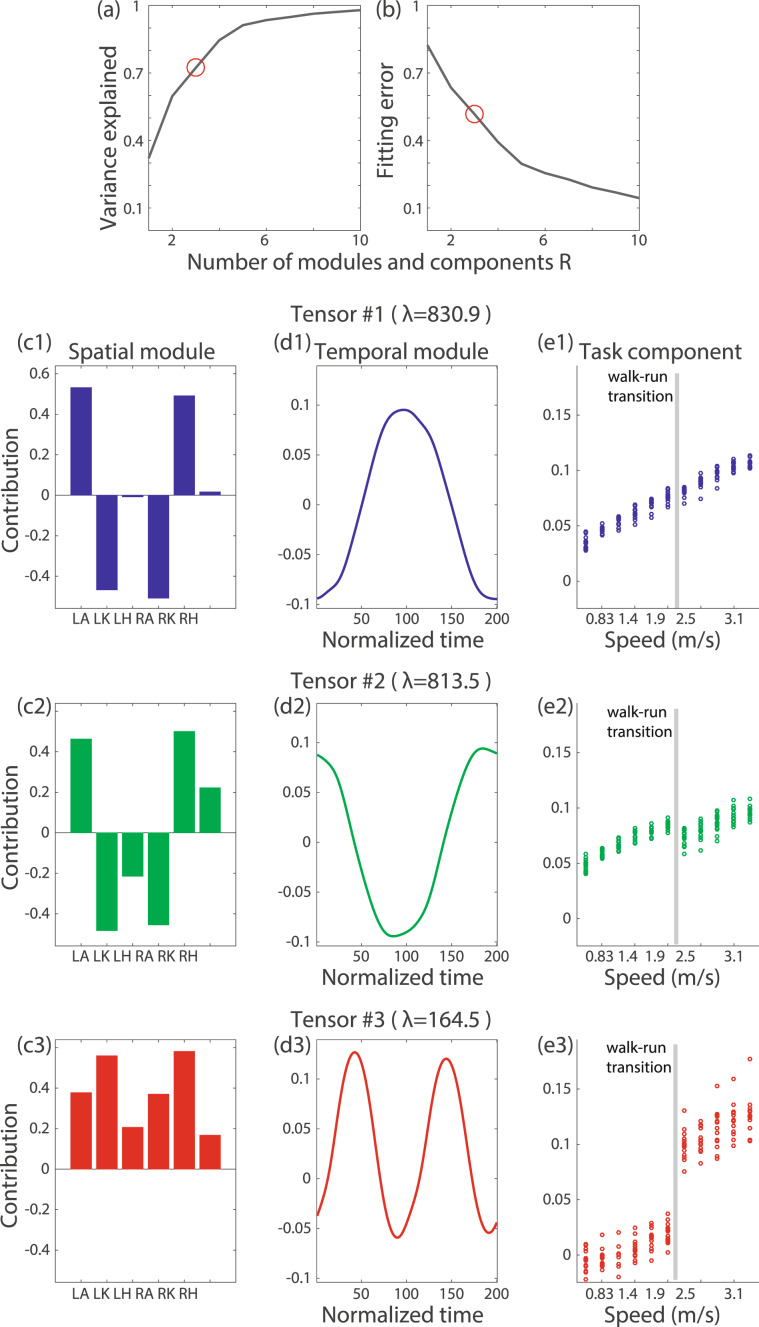
CP decomposition for joint angle data. *λ* denotes the scaling factor for each tensor. (**a**) The relation between the number of modules and components and the variance explained by the tensor decomposition. (**b**) The relation between the number of modules and components and the fitting error of the tensor decomposition. (**c1–c3**) Extracted spatial modules. LA, LK, LH, RA, RK, and RH denote the left ankle, left knee, left hip, right ankle, right knee, and right hip, respectively. (**d1–d3**) Extracted temporal modules. (**e1–e3**) Extracted task-dependent modulations.

**Figure 4:**
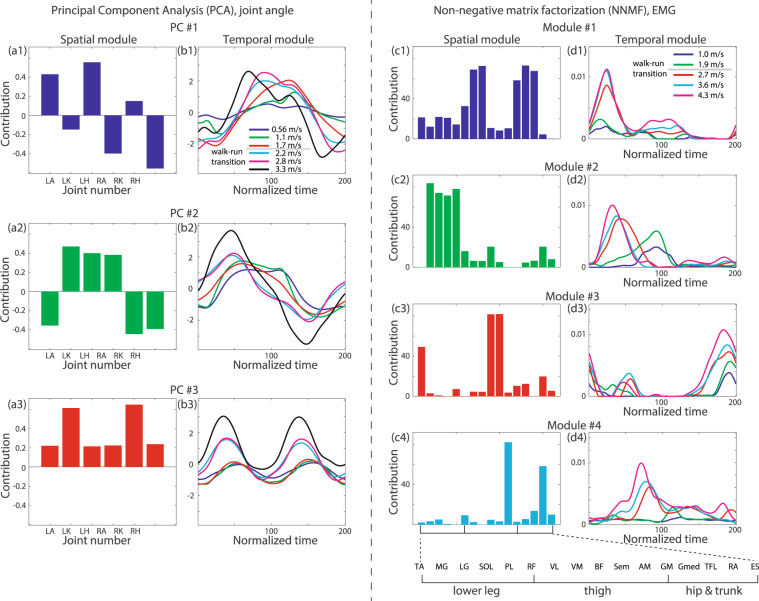
Matrix decompositions for joint angle data and EMG data. (**a1–a3**) Spatial modules extracted via PCA from the joint angle data. LA, LK, LH, RA, RK, and RH denote the left ankle, left knee, left hip, right ankle, right knee, and right hip, respectively. (**b1–b3**) Representative temporal modules extracted via PCA from the joint angle data. The gray horizontal line in b1 indicates the speed range in which all subjects switched from walking to running. (**c1–c4**) Spatial modules extracted via NNMF from the EMG data. The abbreviations for the muscle names are summarized in Table 2. (**d1–d4**) Representative temporal modules extracted via NNMF from the EMG data. The gray horizontal line in d1 indicates the speed range in which all subjects switched from walking to running.

**Figure 5:**
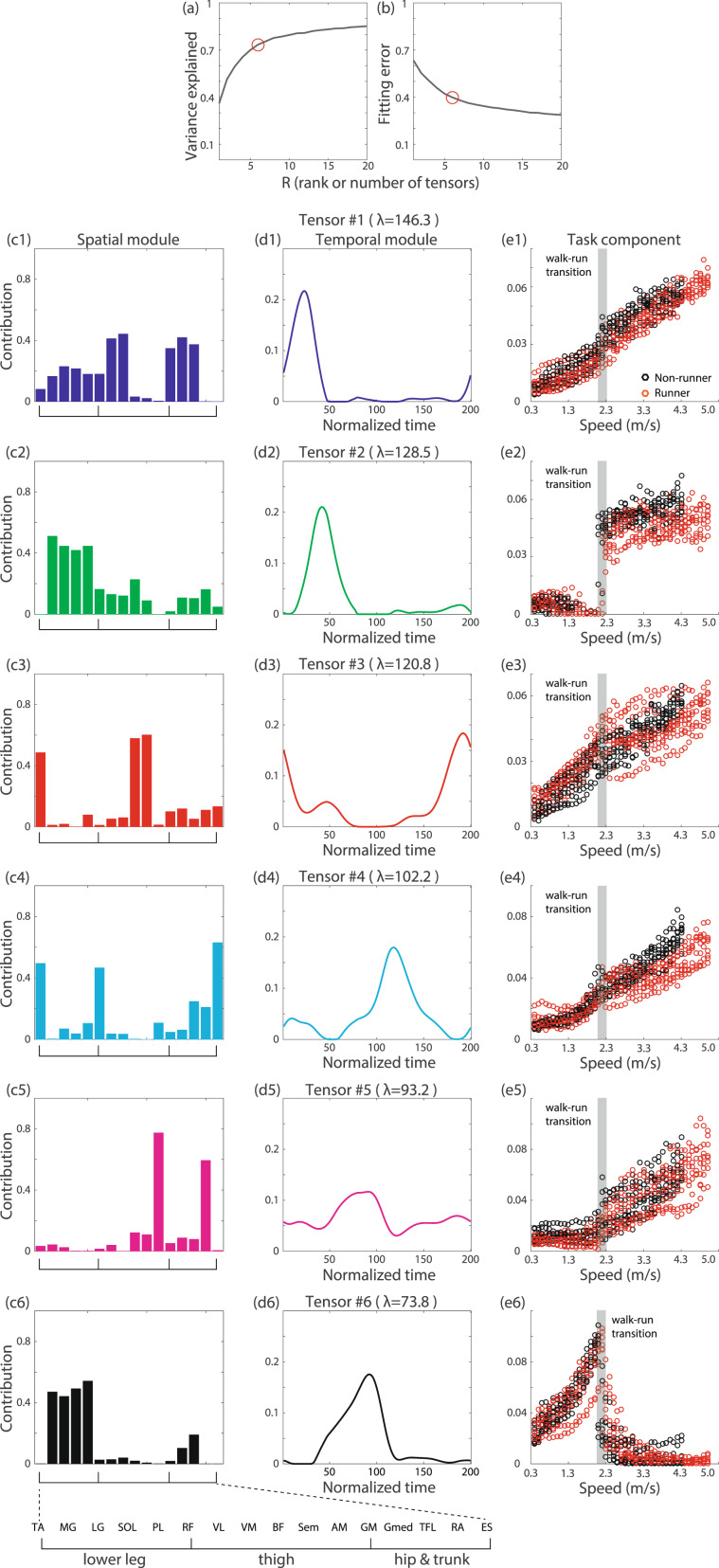
Tensor decomposition for EMG data with non-negativity constraints. *λ* denotes the scaling factor for each tensor. (**a**) The relation between the number of tensors and the variance explained by the tensor decomposition. (**b**) The relation between the number of tensors and the fitting error of the tensor decomposition. (**c1–c6**) Extracted spatial modules. The abbreviations for the muscle names are summarized in Table 2. (**d1–d6**) Extracted temporal modules. (**e1–e6**) Extracted task-dependent modulations.

**Figure 6:**
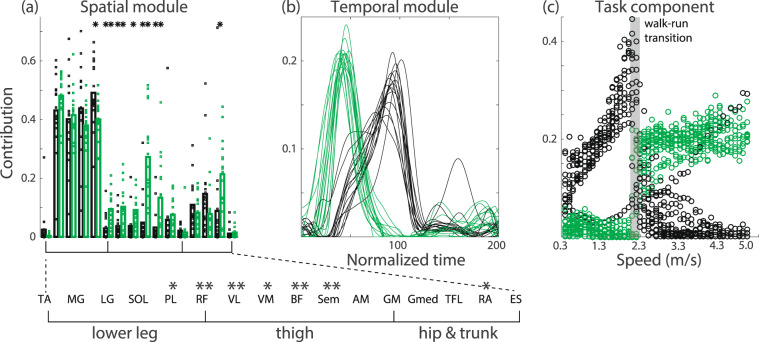
Further analysis of the tensors whose task-dependent modulations show discontinuous changes between walking and running. CP decomposition with non-negativity constraints was applied to the EMG data for each subject. We chose *R* to be the minimum number of modules and components that explained more than 80% of the variance in the original data. Then, we identified the tensors whose task-dependent modulations show the most and second-most substantial discontinuous changes between walking and running. The tensor that shows more significant task-dependent modulation for walking than for running is presented in black, and the tensor that shows more substantial task-dependent modulation for running than for walking is presented in green. (**a**) Spatial modules for each subject. Each dot indicates the recruitment pattern for one spatial module and one subject. Each bar shows the average value of the recruitment pattern across all subjects. Single asterisks and double asterisks associated with certain muscles indicate significant differences in the recruitment patterns between the two tensors, with p < 0.05 and p < 0.01, respectively. The abbreviations of the muscle names are summarized in Table 2. (**b**) Temporal modules for each subject. (**c**) Task-dependent modulations for each subject.

Throughout this study, we choose *R* to be the minimum number of modules that explain more than 70% of the variance in the original data (Figs. 3-5). Equivalently, we choose *R* to be the minimum number required to exceed a value of 0.7 for the uncentered coefficient of determination. This 70% criterion for the analysis of all subjects can be considered to approximately correspond to an 80% criterion in the analysis of individual subjects because these conditions yield similar results (Figs. 5 and 6). Notably, we do not find an intrinsic difference between the *R* values necessary to explain more than 70% and more than 90% of the original variance in the analysis of the joint angle data (Fig. S1) and the values necessary to explain more than 70%, more than 75%, and more than 80% of the original variance in the analysis of the EMG data (Figs. S2–S4). Because the tensor decomposition with *R* = 4 explains more than 70% and that with *R* = 5 explains more than 90% of the original variance in the joint angle analysis, no specific *R* results can be presented to explain more than 80% of the original variance. Although we rely on the variance as the measure for determining *R*, following previous studies using matrix decomposition, we also demonstrate a common measure for determining R in tensor decomposition, with the fitting error defined as $\frac{{\sum }_{i,j,k}\,{({X}_{i,j,k}-{\sum }_{r=1}^{R}{\lambda }_{r}{w}_{i,r}{p}_{j,r}{t}_{k,r})}^{2}}{{\sum }_{i,j,k}\,{X}_{i,j,k}^{2}}$ (Figs. 3b and 5b).

The 70% threshold for the explained variance is lower than the threshold used for matrix decomposition in previous studies^6,8,12^. Notably, the amount of variance explained in CP decomposition is generally lower than that in matrix decomposition due to the number of parameters. In the analysis of *K* conditions, for example, common spatial modules and temporal modules are estimated across all conditions in CP decomposition, as represented in Eq. (2). The fitting power for each condition is controlled by a third factor (i.e., ***t***_*r*_ in the equations mentioned above). In total, the number of fitting parameters is *S* × *R *+ *T* × *R *+ *K* × *R*. In contrast, for matrix decomposition in the form shown in Fig. 1b, the number of fitting parameters is *S* × *R *+ *T* × *K* × *R*. When the data for each condition are analyzed separately, the number of fitting parameters is *S* × *T* × *R* × *K*. In our EMG analysis, *S* is equal to 16, *T* is equal to 200, and *K* is equal to 696. Thus, for CP decomposition, the number of parameters is 912 × *R*, whereas for matrix decomposition, the number of parameters is 139,216 × *R* for the case illustrated in Fig. 1b or 2,227,200 × *R* for the case of separate analysis for each condition. Consequently, the fitting power in matrix decomposition is markedly better than that in CP decomposition due to the large difference in the number of parameters.

In contrast to tensor decomposition, matrix decomposition (e.g., PCA or NNMF) enables the extraction of the spatial modules and the task-dependent modulation of only the temporal modules when we analyze *S* × (*T* × *K*) matrices (Fig. 1b). In the decomposition process, the matrix ***Z*** ∈ **R**^*S*×(*T*×*K*)^ is decomposed as

4 $${\boldsymbol{Z}}\simeq \mathop{\sum }\limits_{r=1}^{R}\,{{\boldsymbol{w}}}_{r}{{\boldsymbol{p}}}_{r}^{T},$$

where *S*, *T*, and *K* denote the number of joint angles or muscles, the number of time frames, and the number of tasks, respectively; *R* is the rank, to be determined *a priori*; ***w***_*r*_ ∈ **R**^*S*×1^ denotes the *r* th spatial module; and ***p***_*r*_ ∈ **R**^(*T*×*K*)×1^ denotes the *r* th temporal module modulated in a task-dependent manner (Fig. 1b). Similar to tensor decomposition, we determine *R* as the minimum number of modules and components that explain more than 70% of the variance in the original data (Fig. 4). In PCA, there are orthogonality restrictions among the spatial modules: ${{w}_{i}}^{T}{{\boldsymbol{w}}}_{{j}}$ = 0 when *i* ≠ *j*. In NNMF, there are no such orthogonality restrictions, but there are non-negativity constraints (i.e., *w*_*i*,*r*_ ≥ 0 and *p*_*j*,*r*_ ≥ 0). After this analysis, we are able to consider how to evaluate the task-dependent modulations of the temporal modules (Fig. 1b). Matrix decomposition can also be separately applied to the *S* × *T* matrix for each task. In that case, however, we generally obtain different spatial and temporal modules for each task; thus, a problem arises in determining how to evaluate the task-dependent modulation of the spatiotemporal modules. A common measure used for this purpose is the pairwise correlation of the spatial modules among the tasks, without considering the task-dependent modulation of the temporal modules^6^. Thus, in matrix decomposition, task-dependent modulation is quantified for either the spatial modules or the temporal modules rather than for both the spatial and temporal modules simultaneously.

In summary, tensor decomposition enables the evaluation of the task-dependent modulations of spatiotemporal modules without being restricted to considering only spatial or only temporal modules.

### Tensor decomposition for joint angle data

The current study focuses on the hip, knee, and ankle angles of the right and left legs in the sagittal plane, as listed in Table 1, during walking or running on a treadmill (Fig. 2 shows the temporal variations of these six angles at six representative speeds). We set 11 different belt speeds and requested participants (N = 15, ages 23–31 years, all male) to walk when the belt speed was 0.56, 0.83, 1.11, 1.39, 1.67, or 1.94 m/s and to run when the belt speed was 2.22, 2.50, 2.78, 3.06, or 3.33 m/s.

**Table 1:** Calculated joint angles and definitions.

Angle	Positive value	Negative value
Ankle	Dorsiflexion	Plantar flexion
Knee	Flexion	Extension
Hip	Flexion	Extension

We applied CP decomposition to the joint angle data of all subjects at all speeds (Fig. 3). We extracted the spatial modules (Fig. 3c), the temporal modules (Fig. 3d), and the task-dependent modulations of those modules (Fig. 3e). Here, the term “task” refers to locomotion at one of the 11 different speeds for one of the 15 subjects—thus, in total, we analyzed 165 “tasks”. Hereafter, we refer to each group consisting of one pair of spatial and temporal modules and the associated task-dependent modulation as a tensor (e.g., an example of a tensor is the combination of a spatial module, a temporal module, and the task-dependent modulation of these modules that is shown in Fig. 1a). An essential consideration in tensor decomposition, or CP decomposition, is that all tensors are unrelated to each other. In other words, the spatial module presented in Fig. 3c1 is associated with the temporal module indicated in Fig. 3d1 and the task-dependent modulation presented in Fig. 3e1; however, that spatial module is not related to any other spatial modules, temporal modules, or task-dependent modulations. Throughout this study, we indicate associated modules and components using the same color, such as blue, green, or red, for CP decomposition. For the case of matrix decomposition (Figs. 1b and 4), color does not always indicate association.

In tensor #1 (Fig. 3c1-f1), the spatial module primarily consists of the left ankle (LA), left knee (LK), right ankle (RA), and right knee (RK). The contributions of the left and right legs are opposite because of their opposite signs in the spatial module. The temporal modulation was minimal when the right foot took off (at time 1) and returned (at time 200) and was maximal when the left foot was on the belt (at approximately time 100). These results indicate that at the contact of the right foot with the belt, the left ankle showed plantar flexion, the left knee showed flexion, the right ankle showed dorsiflexion, and the right knee showed extension. These results similarly indicate that at the contact of the left foot with the belt, the left ankle showed dorsiflexion, the left knee showed extension, the right ankle showed plantar flexion, and the right knee showed flexion. As seen from the task-dependent modulation (Fig. 3e1), this spatiotemporal module was recruited to a greater extent at higher speeds.

In tensor #2 (Fig. 3c2-f2), the left hip (LH) and right hip (RH) are additionally recruited in comparison to tensor #1. The temporal module shows the opposite sign, and the peak timings are slightly different from those in tensor #1. These results indicate that the corresponding temporal variation of the joint angles is opposite to that represented by tensor #1. Tensor #2 was recruited at higher speeds; however, its recruitment slightly and discontinuously decreased when the subject switched from walking to running.

In tensor #3, all joints are cooperatively activated, with two positive and negative peaks in the temporal module. This spatiotemporal module was recruited mainly during running (Fig. 3e3). To our knowledge, although CP decomposition can extract this running-specific spatiotemporal module from joint angle data, PCA cannot.

In summary, CP decomposition enables the extraction of spatial modules, temporal modules, and task-dependent modulations of those modules. To enable a comparison with matrix decomposition, we also applied PCA, a matrix decomposition algorithm mainly used for extracting spatiotemporal modules from joint angle data, to the same data (Fig. 4a,b). When we applied PCA, as demonstrated in Fig. 1b, we obtained common spatial modules across all speeds and subjects (Fig. 4a) and task-dependent modulated temporal modules (Fig. 4b). One difference between tensor decomposition and PCA lies in their orthogonality characteristics. In PCA, the spatial modules are required to be orthogonal to each other; however, this orthogonality restriction originates from mathematical convenience rather than from any true properties of the spatial modules. By contrast, tensor decomposition yields spatiotemporal modules without this restriction. Another difference between tensor decomposition and PCA is the quantification of the task-dependent modulations. In PCA, we need some means of quantifying the task-dependent modulations in the extracted temporal modules shown in Fig. 4b. On the other hand, tensor decomposition can quantify the modulation across all tasks without requiring any *a posteriori* analysis. A simple *a posteriori* analysis approach is to compare the peak values in the temporal modules^8,24^. Although such a comparison can provide useful insights when the peak timings have significant meanings, it is unclear how to evaluate more than two local peaks, especially in the case of Fig. 4b3. It is also unclear how to evaluate the speed-dependent modulation of the temporal modules within the same movement mode (walking or running) (Fig. 4b3). Another simple method of quantifying the task-dependent modulation is to calculate correlation coefficients—correlation analysis can often be applied to generate pairwise similarities. By contrast, tensor decomposition can quantify the task-dependent modulations in a global rather than pairwise manner while considering all tasks simultaneously. This global evaluation enables us to reveal both the linear increase in the recruitment patterns in tensors #1 and #2 and the running-specific recruitment pattern in tensor #3.

### Tensor decomposition for EMG data

Tensor decomposition can be applied not only to joint angle data but also to EMG data with non-negativity constraints. We measured 16 muscles on the right side of the body, listed in Table 2, as 16 subjects (ages 20–31 years, all male) walked or ran on the treadmill. The belt speed was gradually increased from 0.3 to 5.0 m/s for the well-trained college runners (N = 8) and from 0.3 to 4.3 m/s for the non-runners (N = 8). The belt speed was gradually increased following a constant acceleration profile (0.01 m/*s*^2^). The subjects were instructed to walk or run as they chose. As a result, the subjects switched their motion patterns from walking to running within a speed range of approximately 1.9–2.3 m/s. The details of the measured data were described in a previous study^12^.

**Table 2:** Measured muscles and abbreviations.

TA: tibialis anterior	MG: medial gastrocnemius	LG: lateral gastrocnemius	SOL: soleus
PL: peroneus longus	RF: rectus femoris	VL: vastus lateralis	VM: vastus medialis
BF: biceps femoris (long head)	ST: semitendinosus	AM: adductor magnus	GM: gluteus maximus
Gmed: gluteus medius	TFL: tensor fasciae latae	RA: rectus abdominis	ES: erector spinae

We applied tensor decomposition and extracted six tensors (Fig. 5). Each tensor has a different functional role. In tensor #1 (Fig. 5c1-f1), all muscles in the lower legs, the quadriceps muscles, and all hip muscles are activated (Fig. 5c1) upon contact of the right foot (Fig. 5d1). This spatiotemporal module was recruited to a greater extent at higher speeds (Fig. 5e1), as identified based on the quantification of the task-dependent modulation of the extracted modules enabled by CP decomposition. Similar task-dependent modulations can be observed in tensors #3, #4, and #5. Despite the similarities in the task-dependent modulations, the spatial and temporal modules in these tensors differ from those in tensor #1.

In tensors #2 and #6, the task-dependent modulations show discontinuous changes between walking and running (Fig. 5e2,e6); tensor #2 appears to be related to running, tensor #6 appears to be related to walking. Because these tensors likely provide the neural mechanisms facilitating switching between the two modes, we further investigated the properties of these tensors.

To investigate tensors #2 and #6 in detail, we applied tensor decomposition to the EMG data for each subject individually. We chose *R* to be the minimum number of modules and components that explained more than 80% of the variance in the original data. With this criterion, the most suitable *R* value was estimated to be six across all subjects, in agreement with the *R* value estimated for the analysis performed across all subjects simultaneously (Fig. 5). After identifying the two tensors whose task-dependent modulations show the largest and second largest changes between walking and running, we plotted the corresponding spatial modules (Fig. 6a), temporal modules (Fig. 6b), and task-dependent modulations (Fig. 6c). The task-dependent modulations show discontinuous changes between walking and running. In particular, the tensor whose properties are shown in black was recruited mainly for walking, and the tensor whose properties are shown in green was recruited mainly during running (Fig. 6c). Regarding the temporal modules, the peak timings are different between these tensors, supporting the previous hypothesis reported on the basis of matrix decomposition^4,24^. In addition, different spatial modules were extracted through CP decomposition (Fig. 6a). A single asterisk denotes a significant difference with p < 0.05, and double asterisks denote a significant difference with p < 0.01 (p = 2.36 × 10^−9^ [F(15,225) = 5.44] for the interaction between the muscle factor and the mode factor [walking or running], with p = 0.0200 for PL, p = 0.00847 for RF, p = 0.00987 for VL, p = 0.0318 for VM, p = 0.000218 for BF, p = 0.00990 for ST, and p = 0.0416 for RA). The details of the statistical analysis are given in the Methods section. Several significant differences are evident between the spatial modules related to walking and running. In particular, the thigh muscles are more activated in the spatial module that is recruited mainly in running than in the spatial module that is recruited mainly in walking. Although the higher recruitment of the thigh muscles in running compared with walking seems evident from the analysis of each single muscle, it is unclear from this analysis whether similar recruitment patterns also appear in the related spatial modules. CP decomposition enables us to clarify the involvement of the modulation of thigh muscle activity in the spatial modules while globally comparing locomotion at several speeds.

One difference between CP decomposition and NNMF is how task-dependent modulation is quantified. Via NNMF (Fig. 4c,d), we obtained common spatial modules across all tasks and temporal modules modulated in a task-dependent manner. NNMF requires some *a posterior* analysis to evaluate the modulation. A simple *a posteriori* analysis approach is to compare the peak values in the temporal modules^8,24^. Although such a comparison can provide useful insights when the peak timings have significant meanings, it is unclear how to evaluate subtle peaks, such as in the cases of Fig. 4d1,d4. The peak timings enable us to distinguish between walking and running; however, it is again unclear how to evaluate the speed-dependent modulation of the temporal modules within the same mode (walking or running) (Fig. 4d2). Another popular method is to utilize correlation coefficients. Although this method is convenient, it has limitations; correlation coefficients are often suitable only for evaluating local pairwise relations. Moreover, NNMF in the form illustrated in Fig. 1b assumes common spatial modules across all tasks. By contrast, tensor decomposition enables us to globally quantify the task-dependent modulations of spatiotemporal modules without requiring any *a posteriori* analysis.

## Discussion

The current study has demonstrated the effectiveness of CP decomposition, a tensor decomposition method, for analyzing the task-dependent modulations of spatiotemporal modules extracted from joint angle data (Fig. 3) and EMG data with non-negativity constraints (Fig. 5). CP decomposition is closely related to dynamic motor primitives^25^ and enables the extraction of time-varying motor commands (i.e., temporal modules) sent to groups of muscles (i.e., spatial modules). Although the temporal widths of these time-varying motor commands were addressed by means of temporal normalization in the current study, these widths can be modulated appropriately via the systems responsible for controlling movement time in the framework of dynamic motor primitives. Matrix decomposition methods, such as PCA and NNMF, are a popular approach for quantifying spatial modules, temporal modules, and task-dependent modulations of either spatial or temporal modules when combined with *a posteriori* analysis^7–9,11,12^. By contrast, as shown in this study, tensor decomposition enables the quantification of task-dependent modulations in both spatial and temporal modules simultaneously, with little *a posteriori* analysis required (Figs. 3, 5 and 6). Additional statistical analyses provide further information about the neural control of walking and running movements (Fig. 6). Tensor decomposition can thus be used to evaluate the task-dependent modulations of spatiotemporal modules in a straightforward manner.

The current study simultaneously focused on both the task- and subject-dependent modulations of spatiotemporal modules. Tensor decomposition considering a third (“task”) factor enabled us to quantify how each spatiotemporal module was recruited at each speed in each subject. In general, tensor decomposition can enable the consideration of more than three factors; thus, it would also be possible to define speed as the third factor and subject as the fourth factor. In such a four-factor analysis, we could investigate how each combination of a spatial module, a temporal module, and the task-dependent modulation of these modules is recruited in each subject. In other words, common combinations would be extracted for all subjects to evaluate the recruitment pattern in each subject. For example, if one subject switched from walking to running at approximately 2.0 m/s and another subject performed the walk-run transition at approximately 2.1 m/s, higher fitting performance could be achieved in tensor decomposition by either fitting subject-specific combinations or neglecting the data near the speed range of the walk-run transition. In the case of fitting subject-specific combinations, the four-factor analysis approach would not be as meaningful; we would need to analyze the data for each subject separately to discuss task-dependent modulation. In the case of neglecting the data near the speed range of the walk-run transition, we would not be able to evaluate the task-dependent modulations of the spatiotemporal modules in this speed range. In our experimental setting, the subjects showed different walk-run transition speeds during the measurement of the EMG data. We thus would be likely to overlook some aspects of the task-dependent modulations in a four-factor analysis.

In contrast to a four-factor analysis, the current three-factor analysis allows us to evaluate the “task”-dependent modulations of the spatiotemporal modules while considering diverse individual differences in speed for the walk-run transition. In this case, the “task” dimension (the third dimension) includes both the task and subject factors. This analysis thus enables the evaluation of how each spatiotemporal module is recruited in each task and subject while considering individual differences in the walk-run transition. Because these individual differences were smaller than the speed-dependent modulations between walking and running in our experimental setting, we obtained similar results in the analysis of all subjects together (Fig. 5) and each subject individually (Fig. 6). The current three-factor analysis enabled us to clarify how the spatiotemporal modules were modulated with respect to speed. To focus on the details of the task-dependent modulations, it was necessary to analyze the tensor data collected under all conditions for each subject individually, such as in the analysis of the EMG data (Fig. 6). On the other hand, if we wished to focus on the details of the individual differences, we would need to analyze the tensor data collected for all subjects under each condition individually. Due to the flexibility of the tensor decomposition approach, it is necessary to carefully structure the tensor data in accordance with the main purpose of the analysis.

Following the proper selection of the third factor to be considered in the analysis (in addition to the spatial and temporal factors), CP decomposition enables the investigation of several features inherent in joint angle and EMG data. A promising possibility is to utilize CP decomposition to investigate individual differences. For example, for comparing the walking patterns of young and elderly people, the third dimension can be subject number. In this case, the third factor *t*_*k*,*r*_ can illustrate how different spatiotemporal modules are recruited between young and elderly walkers. Another promising possibility is to apply CP decomposition to investigate task-dependent modulations in various kinds of walking and running, such as walking on a slippery surface, race walking, or walking with one’s eyes closed. One potential approach is to apply CP decomposition to joint angle or EMG data recorded in response to perturbations^8,26^, which would enable the evaluation of how spatiotemporal modules are recruited to compensate for perturbations. A third promising possibility is to apply CP decomposition to kinematic and EMG data related to motor adaptation, motor learning, development, or rehabilitation^27–29^. In this case, the third factor can be the trial number or day. In this case, the third factor *t*_*k*,*r*_ can illustrate how spatiotemporal modules are modulated depending on adaptation, learning, development, or rehabilitation. Because several studies have focused on both adaptation^27–35^ and spatiotemporal modules^2–6^ in detail but only separately, the relationship between those concepts has been investigated in only a few studies^10,16^. Investigating the link between motor adaptation and spatiotemporal modules via tensor decomposition may be a promising direction for future work.

The proper way to determine the number of modules depends on the user requirements. There is no unified method of doing so, as in the case of matrix decomposition^15^. The only general requirement is that the number of modules should not be set so high that they explain almost 100% of the variance. In such an overfitted scenario, the fitted results will include noise in many cases^15^. Notably, our current findings are invariant across several conditions (see Figs. 3, 5, and S1–S4). Using various criteria, we confirmed (1) the linear increases in the recruitment patterns of the spatiotemporal modules in proportion to speed, independent of the movement mode (walking or running) (see, e.g., Figs. 3e1,e2, 5c1,c3,c4, S1c1, S1c2, S2c2, S3c1,and S3c3); (2) the discontinuous change in the recruitment patterns when switching between walking and running (see, e.g., Figs. 3e2,e3, 5e2,e6, S1c3, S1c4, S2c3, S2c6, S3c2, and S4c8); and (3) the low recruitment of thigh muscles in walking-specific spatiotemporal modules and the high recruitment of thigh muscles in running-specific modules (see, e.g., Figs. 5c2,c6, S2a3, S2a6, S3a2 and S4a8).

Another variant of matrix decomposition has been proposed in a previous study^5^. This sophisticated method enables the consideration of spatiotemporal modules without separating the spatial and temporal aspects. The separation of the spatial and temporal modules that is inherent in PCA, NNMF, and tensor decomposition enables the examination of the temporal variations of groups of muscles without any time delays among individual muscles. In other words, these methods enable the consideration of simultaneous and synchronous activities among multiple muscles. On the other hand, the method proposed in the previous study^5^ enables the consideration of delayed synchronous activities among multiple muscles. A promising direction for future work is to combine the advantages of the method proposed in the previous study^5^ with the advantages of tensor decomposition to evaluate the task-dependent modulations of spatiotemporal modules while considering delayed synchronization.

CP decomposition is the simplest version of tensor decomposition; it would also be possible to apply a more sophisticated version of tensor decomposition. A popular alternative is Tucker decomposition^15^, a variant of which has previously been applied to EMG data^17–21^. In Tucker decomposition, the number of spatial modules, the number of temporal modules, and the number of task-dependent modulations can differ from each other. Notably, CP decomposition is a special case of Tucker decomposition; thus, Tucker decomposition is more general. On the other hand, Tucker decomposition involves three free parameters (i.e., the number of spatial modules, the number of temporal modules, and the number of task-dependent modulations) and consequently consumes considerably more computational time than CP decomposition. We should note that Tucker decomposition can be a powerful tool for analyzing some complex data because of its own intrinsic complexity^36^. However, CP decomposition was suitable for the current study because relatively few complicated combinations of spatiotemporal modules are related to locomotion.

Another possible variant of tensor decomposition is to include a smoothness property^37^. Because the temporal variations of joint angle and EMG data are smooth, the smoothness property can be used to effectively denoise these data, such as in the state space model^38–41^. For the analysis of a single condition and subject, the smoothness property can also be effectively applied to task-dependent modulations.

## Materials and Methods

### Ethics statement

A total of 31 healthy volunteers (ages 20–31 years, all male) participated in our experiments, which were approved by the ethics committee of the University of Tokyo and were performed following the relevant guidelines and regulations. All participants were informed of the experimental procedures following the Declaration of Helsinki, and all participants provided written informed consent before the start of the experiments.

### Experimental setup, data acquisition, and data processing (joint angles)

A total of 15 participants participated in our experiment for measuring joint angles. They performed walking at six speeds (0.56, 0.83, 1.11, 1.39, 1.67, and 1.94 m/s) and running at five speeds (2.22, 2.50, 2.78, 3.06, and 3.33 m/s) on a treadmill (Bertec, Columbus, OH, USA). Under all conditions, we measured more than 27 strides; we thus analyzed the joint angles averaged across the first 27 strides for all speeds and subjects.

The joint angles were recorded at 100 Hz using 12 cameras (OptiTrack V100:R2, NaturalPoint Inc., Corvallis, Oregon). The measured marker positions were low-pass filtered with a zero-lag Butterworth filter (15-Hz cut-off, 4th order) and transformed into joint angles in the sagittal plane (i.e., right and left ankle, knee, and hip angles).

Three-dimensional ground reaction force (GRF) data were recorded at 1000 Hz by force plates under each belt of the treadmill. The sampling rate was modified to 100 Hz to match the rate of the joint angles. The GRF data were low-pass filtered with a zero-lag Butterworth filter (15-Hz cut-off, 4th order). The times corresponding to foot contact and toe-off were determined on a stride-by-stride basis from the vertical component of the GRF.

Because the stride-to-stride cycle differed depending on the speed, we normalized all cycles to 200 time frames for all speeds and subjects. In addition, we standardized the joint angles such that the mean and standard deviation of each angle for each subject across all speeds were 0 and 1, respectively. These normalization and standardization procedures enabled us to compare different joint angles, speeds, and subjects fairly. In total, the joint angle data included six joint angles, 200 time frames, and 11 × 15 = 165 tasks (i.e., the number of speeds × the number of subjects). Throughout this study, the word “task” broadly refers to locomotion at a particular speed for a particular subject. In this case, the 165 tasks included 11 speeds (i.e., 11 types of tasks) and 15 subjects. We thus constructed a data tensor ***X*** ∈ **R**^6×200×165^ to apply tensor decomposition to all data simultaneously.

### Experimental setup, data acquisition, and data processing (EMG)

We also analyzed EMG data that we had collected in a previous study, the details of which can be found in^12^. A total of 16 participants participated in our experiment for measuring EMG data. A total of 8 of the sixteen participants were well-trained college runners. The runners were asked to move at higher speeds than the other participants. All participants walked or ran on the same treadmill, as mentioned above, with a linearly increasing speed (speed ramping conditions with the acceleration set to 0.01 m/*s*^2^). The speed range was adjusted to be safe for each group but to vary as widely as possible (0.3–4.3 m/s for the non-runner group and 0.3–5.0 m/s for the runner group). The participants were instructed to choose to either walk or run depending on their preference at the given speed. The speed of the transition from walking to running for all participants ranged from 1.9 to 2.3 m/s. Because the acceleration was low and the maximum speeds were considered safe for each group, the locomotive movements performed by all participants were always stable throughout the experiment.

Three-dimensional GRF data were recorded in the same manner described above. Surface EMG activity was recorded from the 16 muscles listed in Table 2 on the right side of the trunk and leg. The EMG activity was recorded with a wireless EMG system (Trigno Wireless EMG System; Delsys, Boston, MA, USA). The EMG signals were bandpass filtered (20–450 Hz), amplified (with a 300 gain preamplifier), and sampled at 1000 Hz. The EMG data were digitally full-wave rectified and smoothed and were also low-pass filtered with a zero-lag Butterworth filter.

Because the stride-to-stride cycle differed depending on the speed, we normalized all cycles to 200 time frames for all speeds and subjects. Accordingly, we normalized all of the EMG signals. In addition, we scaled the EMG signals such that the maximum value for each muscle and each subject across all speeds was 1. In the analysis, we divided the belt speed into 0.1 m/s intervals, e.g., 0.3–0.4 m/s and 0.4–0.5 m/s. After defining the speed ranges, we averaged the EMG activity in each speed range. In total, the EMG data for the non-runners consisted of 16 muscles, 200 time frames, and 40 × 8 tasks (i.e., 40 speed ranges and eight subjects). The EMG data for the runners consisted of 16 muscles, 200 time frames, and 47 × 8 tasks (i.e., 47 speed ranges and eight subjects). We thus constructed a data tensor ***X*** ∈ **R**^16×200×696^ to apply tensor decomposition to all data simultaneously. For the application of tensor decomposition to the data for each subject individually, the size of the data tensor was ***X*** ∈ **R**^16×200×40^ for each non-runner and ***X*** ∈ **R**^16×200×47^ for each runner. When applying tensor decomposition to the EMG data for each subject individually (Fig. 4), we set the number of tensors *R* to 6 to keep this number the same as that used in the analysis of all subjects (Fig. 3).

### Tensor decomposition

We relied on the tensor toolbox in MATLAB^42,43^ and used the function “cp_als” (alternating least squares^15^) to analyze the joint angles and the function “cp_nmu” (multiplicative update, similar to NNMF^14^) to analyze the EMG signals.

An important aspect of the tensor decomposition process for the joint angle data (i.e., data without non-negativity constraints) is that any two pairs of components can be reversed in sign but have the same approximate value. For example, when ***w***_r_ →−*** w***_*r*_ and ***p***_*r*_ →−*** p***_*r*_, the approximate values remain invariant. When tensor decomposition is applied to the joint angle data of two subjects separately, the signs of the spatiotemporal modules may be opposite even if the modules are actually similar for each subject. We therefore applied tensor decomposition to the joint angle data for all subjects simultaneously to estimate common spatiotemporal modules and task-dependent modulations of these modules for each speed and each subject.

### Matrix decomposition

For comparing tensor decomposition with PCA and NNMF, we relied on the MATLAB functions “pca” and “nnmf”.

For the application of matrix decomposition to the joint angle data, the size of the data matrix was ***Z*** ∈ **R**^6×16500^, where 6 is the number of joints and 33000 is the product of the number of time frames, the number of speeds, and the number of participants. For the application of NNMF to the EMG signals, the size of the data matrix was ***Z*** ∈ **R**^16×139400^, where 16 is the number of muscles and 139400 is the product of the number of time frames, the number of speed ranges, and the number of participants.

### Statistical test

To compare the task-dependent modulations of the two representative tensors in Fig. 6, we first extracted the two tensors of interest based on the absolute difference in the task-dependent modulations between walking (at 1.8 m/s) and running (at 2.3 m/s). For the case in which the task-dependent modulation was larger for walking than for running, all corresponding components are shown in black. For the case in which the task-dependent modulation was greater for running than for walking, all corresponding components are shown in green. The choice of these colors is based on the results depicted in Fig. 5. Tensors #2 (green) and #6 (black) in Fig. 5 exhibit task-dependent modulations that are larger for running than for walking and larger for walking than for running, respectively.

After separating these two tensors, we performed repeated-measure ANOVA on the recruitment values in the spatial modules considering two factors: the specific muscle and the movement mode (walking or running). After confirming the interaction between those factors, we performed Tukey’s post hoc test.

## Supplementary information

Supplementary Information.

## Data Availability

The datasets analyzed in the current study are available from the corresponding author upon reasonable request.

## References

[1] Bernstein N. A. The coordination and regulation of movements. Pergamon, London (1967).

[2] Bizzi E, Mussa-Ivaldi FA, Giszter S (1991). Computations underlying the execution of movement: a biological perspective. Science.

[3] Borghese NA, Bianchi L, Lacquaniti F (1996). Kinematic determinants of human locomotion. J. Physiol..

[4] Ivanenko YP, Poppele RE, Lacquaniti F (2004). Five basic muscle activation patterns account for muscle activity during human locomotion. J. Physiol..

[5] d’Avella A, Saltiel P, Bizzi E (2003). Combinations of muscle synergies in the construction of a natural motor behavior. Nat. Neurosci..

[6] Torres-Oviedo G, Ting LH (2007). Muscle synergies characterizing human postural responses. J. Neuro-physiol..

[7] Ivanenko YP, Cappellini G, Dominici N, Poppele RE, Lacquaniti F (2005). Coordination of Locomotion with Voluntary Movements in Humans. J. Neurosci..

[8] Chvatal SA, Ting LH (2012). Voluntary and reactive recruitment of locomotor muscle synergies during perturbed walking. J. Neurosci..

[9] Torres-Oviedo G, Ting LH (2010). Subject-specific muscle synergies in human balance control are consistent across different biomechanical contexts. J. Neurophysiol..

[10] Berger DJ, Gentner R, Edmunds T, Pai DK, d’Avella A (2013). Differences in Adaptation Rates after Virtual Surgeries Provide Direct Evidence for Modularity. J. Neurosci..

[11] d’Avella A, Bizzi E (2005). Shared and specific muscle synergies in natural motor behaviors. Proc. Natl. Acad. Sci..

[12] Yokoyama H, Ogawa T, Kawashima N, Shinya M, Nakazawa K (2016). Distinct sets of locomotor modules control the speed and modes of human locomotion. Sci. Rep..

[13] Bishop C. M. Pattern Recognition and Machine Learning. Springer Verlag. (2006).

[14] Lee DD, Seung HS (1999). Learning the parts of objects by non-negative matrix factorization. Nature.

[15] Kolda TG, Bader BW (2009). Tensor Decompositions and Applications. SIAM Rev..

[16] Williams AH (2018). Unsupervised Discovery of Demixed, Low-Dimensional Neural Dynamics across Multiple Timescales through Tensor Component Analysis. Neuron.

[17] Ebied A, Kinney-Lang E, Spyrou L, Escudero J (2019). Muscle Activity Analysis using Higher-Order Tensor Decomposition: Application to Muscle Synergy Extraction. IEEE Access.

[18] Delis I, Panzeri S, Pozzo T, Berret B (2014). A unifying model of concurrent spatial and temporal modularity in muscle activity. J. Neurophysiol..

[19] Delis I, Panzeri S, Pozzo T, Berret B (2015). Task-discriminative space-by-time factorization of muscle activity. Front. Hum. Neurosci..

[20] Delis I, Hilt PM, Pozzo T, Panzeri S, Berret B (2018). Deciphering the functional role of spatial and temporal muscle synergies in whole-body movements. Sci. Rep..

[21] Hilt PM, Delis I, Pozzo T, Berret B (2018). Space-by-time modular decomposition effectively describes whole-body muscle activity during upright reaching in various directions. Front. Comput. Neurosci..

[22] Schwartz MH, Rozumalski A, Trost JP (2008). The effect of walking speed on the gait of typically developing children. J. Biomech..

[23] Dillmann U (2014). Principal Component Analysis of gait in Parkinson’s disease: relevance of gait velocity. Gait. & Posture.

[24] Cappellini G, Ivanenko YP, Poppele RE, Lacquaniti F (2006). Motor patterns in human walking and running. J. Neurophysiol..

[25] Ijspeert AJ, Nakanishi J, Hoffman H, Pastor P, Schaal S (2013). Dynamical Movement Primitives: Learning Attractor Models for Motor Behaviors. Neural Comput..

[26] Shinya M, Kawashima N, Nakazawa K (2016). Temporal, but not Directional, Prior Knowledge Shortens Muscle Reex Latency in Response to Sudden Transition of Support Surface During Walking. Front. Hum. Neurosci..

[27] Shadmehr R, Mussa-Ivaldi FA (1994). Adaptive representation of dynamics during learning of a motor task. J. Neurosci..

[28] Thoroughman KA, Shadmehr R (2000). Learning of action through adaptive combination of motor primitives. Nature.

[29] Choi JT, Bastian AJ (2007). Adaptation reveals independent control networks for human walking. Nature.

[30] Furuki D, Takiyama K (2019). Decomposing motion that changes over time into task-relevant and task-irrelevant components in a data-driven manner: application to motor adaptation in whole-body movements. Sci. Rep..

[31] Takiyama K (2015). Context-dependent memory decay is evidence of effort minimization in motor learning: a computational study. Front Comput Neurosci.

[32] Takiyama K, Hirashima M, Nozaki D (2015). Prospective errors determine motor learning. Nat. Comm..

[33] Takiyama K, Sakai Y (2016). Balanced motor primitive can explain generalization of motor learning effects between unimanual and bimanual movements. Sci. Rep..

[34] Ishii K, Hayashi T, Takiyama K (2018). Inuence of switching rule on motor learning. Sci. Rep..

[35] Takiyama K, Shinya M (2016). Development of Portable Motor Learning Laboratory (PoMLab). PLoS ONE.

[36] Onken A (2016). Using Matrix and Tensor Factorizations for the Single-Trial Analysis of Population Spike Trains. PLoS Comput. Biol..

[37] Imaizumi M. & Hayashi K. Tensor Decomposition with Smoothness. *Proc. the 34th Int. Conf. on Mach. Learn* 1597–1606 (2017)

[38] Roweis S, Ghahramani Z (1999). A unifying review of linear gaussian models. Neural. Netw..

[39] Takiyama K, Katahira K, Okada M (2009). Exact inference in discontinuous firing rate estimation using belief propagation. J. Phy. Soc. Jpn..

[40] Takiyama K, Okada M (2011). Detection of hidden structures in nonstationary spike trains. Neural. Netw..

[41] Naruse Y, Takiyama K, Okada M, Umehara H (2013). Statistical method for detecting phase shifts in alpha rhythm from human electroencephalogram data. Phys. Rev. E., Statistical, nonlinear, and soft matter physics.

[42] Bader B. W. Others, MATLAB Tensor Toolbox Version **3**.0 (2017).

[43] Bader BW, Kolda TG (2006). Algorithm 862: MATLAB tensor classes for fast algorithm prototyping. ACM Trans. Math Soft..

