# Author Correction: Speed-dependent and mode-dependent modulations of spatiotemporal modules in human locomotion extracted via tensor decomposition

**DOI:** 10.1038/s41598-020-60884-9

**Published:** 2020-03-06

**Authors:** Ken Takiyama, Hikaru Yokoyama, Naotsugu Kaneko, Kimitaka Nakazawa

**Affiliations:** 1 Tokyo University of Agriculture and Technology, Department of Electrical Engineering and Computer Science, Nakacho, Koganei, Tokyo Japan; 2 Rehabilitation Engineering Laboratory, Toronto Rehabilitation Institute, University Health Network, Toronto, Ontario Canada; 3 The University of Tokyo, Department of Life Sciences, Graduate School of Arts and Sciences, Tokyo, Japan

Correction to: *Scientific Reports* 10.1038/s41598-020-57513-w, published online 20 January 2020

The original version of this Article contained an error in the title of the paper, where the words “spatiotemporal” and “decomposition” were incorrectly given as “spatiotem-poral” and “decom-position”. This has now been corrected in the PDF and HTML versions of the Article.

