# Author Correction: Speed-dependent and mode-dependent modulations of spatiotemporal modules in human locomotion extracted via tensor decomposition

**DOI:** 10.1038/s41598-022-21615-4

**Published:** 2022-11-02

**Authors:** Ken Takiyama, Hikaru Yokoyama, Naotsugu Kaneko, Kimitaka Nakazawa

**Affiliations:** 1 Tokyo University of Agriculture and Technology, Department of Electrical Engineering and Computer Science, Nakacho, Koganei, Tokyo Japan; 2 Rehabilitation Engineering Laboratory, Toronto Rehabilitation Institute, University Health Network, Toronto, Ontario Canada; 3 The University of Tokyo, Department of Life Sciences, Graduate School of Arts and Sciences, Tokyo, Japan

Correction to: *Scientific Reports* https://doi.org/10.1038/s41598-020-57513-w, published online 20 January 2020

The original version of this Article contained errors in the Introduction. As a result,

“How the central nervous system (CNS) controls the human body is a fundamental question. The human body possesses 360 joints and more than 650 muscles; the CNS controls the body while somehow resolving a significant number of degrees of freedom (DoFs), in fact, more than are necessary to achieve the desired motions^1^. For example, during walking and running in daily life, these large numbers of joints and muscles should be controlled in an orchestrated manner.”

now reads:

“How the central nervous system (CNS) controls the human body is a fundamental question. The CNS controls the body while somehow resolving a significant number of degrees of freedom (DoFs), in fact, more than is necessary to achieve the desired motions^1^. For example, during walking and running in daily life, large numbers of joints and muscles should be controlled in an orchestrated manner.”

The original Article has been corrected.

