# Supplementary material for: Speed-dependent and mode-dependent modulations of spatiotemporal modules in human locomotion extracted via tensor decomposition
Source: Sci Rep. 2020 Jan 20;10:680. doi: 10.1038/s41598-020-57513-w (PMC6971295; doi:10.1038/s41598-020-57513-w)
Supplement: Supplementary file 1 — Supplementary Information. [file 41598_2020_57513_MOESM1_ESM.pdf]

Supplementary materials for “Speed-dependent and mode-dependent modulations of spatiotemporal modules in human locomotion extracted via tensor decomposition”

Ken Takiyama[1]*, Hikaru Yokoyama[1, 2]*, Naotsugu Kaneko[3], Kimitaka Nakazawa[3]
[1]Tokyo University of Agriculture and Technology, Department of Electrical Engineering and Computer Science, Nakacho, Koganei Tokyo, Japan
[2]Rehabilitation Engineering Laboratory, Toronto Rehabilitation Institute, University Health Network, Toronto, Ontario, Canada
[3]The University of Tokyo, Department of Life Sciences, Graduate School of Arts and Sciences, Tokyo, Japan
* ... equally contributed.
Correspondence should be addressed to K.T. (t.j.ken.takiyama@gmail.com).

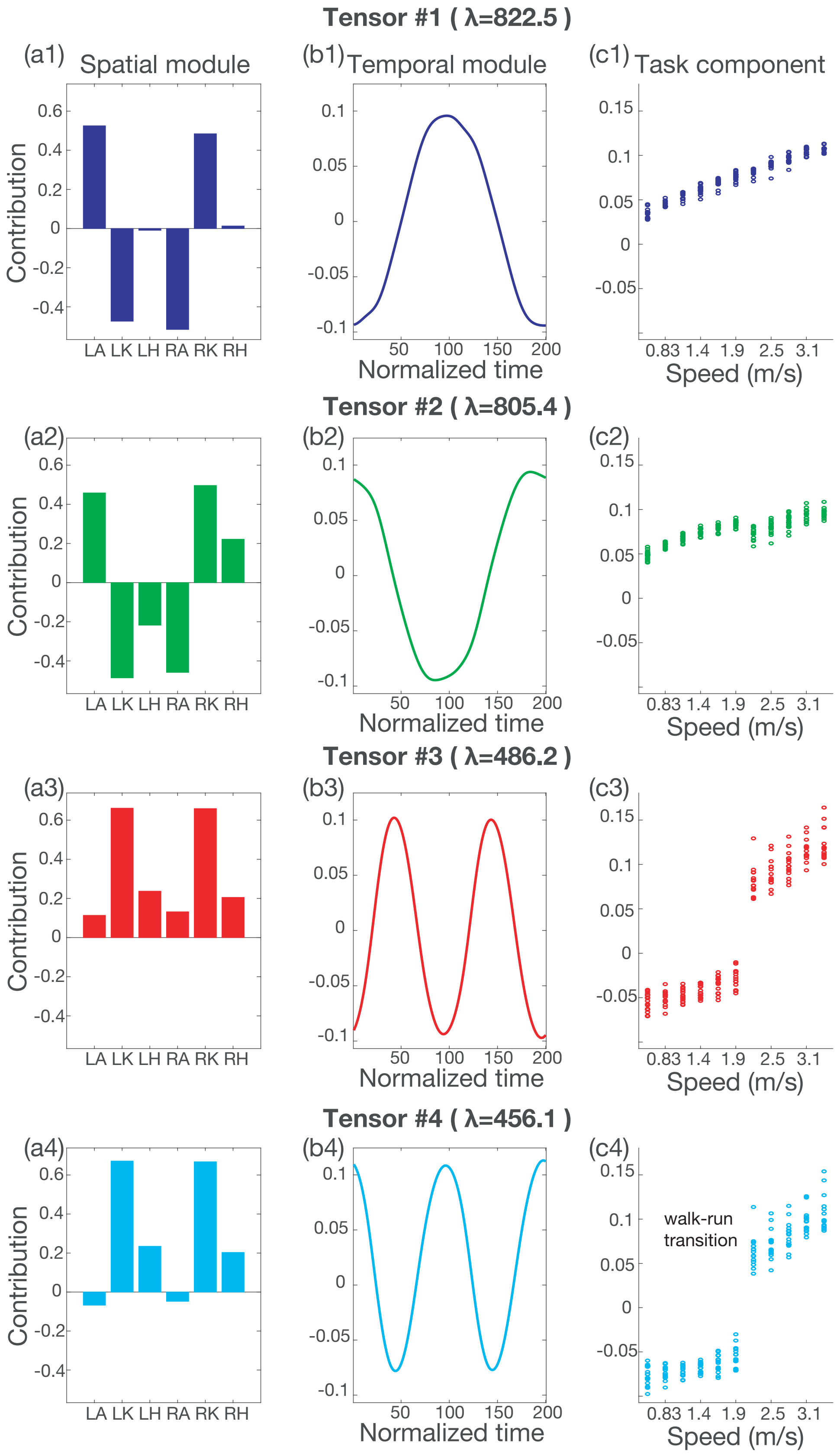

Fig. S1 Tensor decomposition for joint angle data. The number of modules was determined based on 90% explained variance.

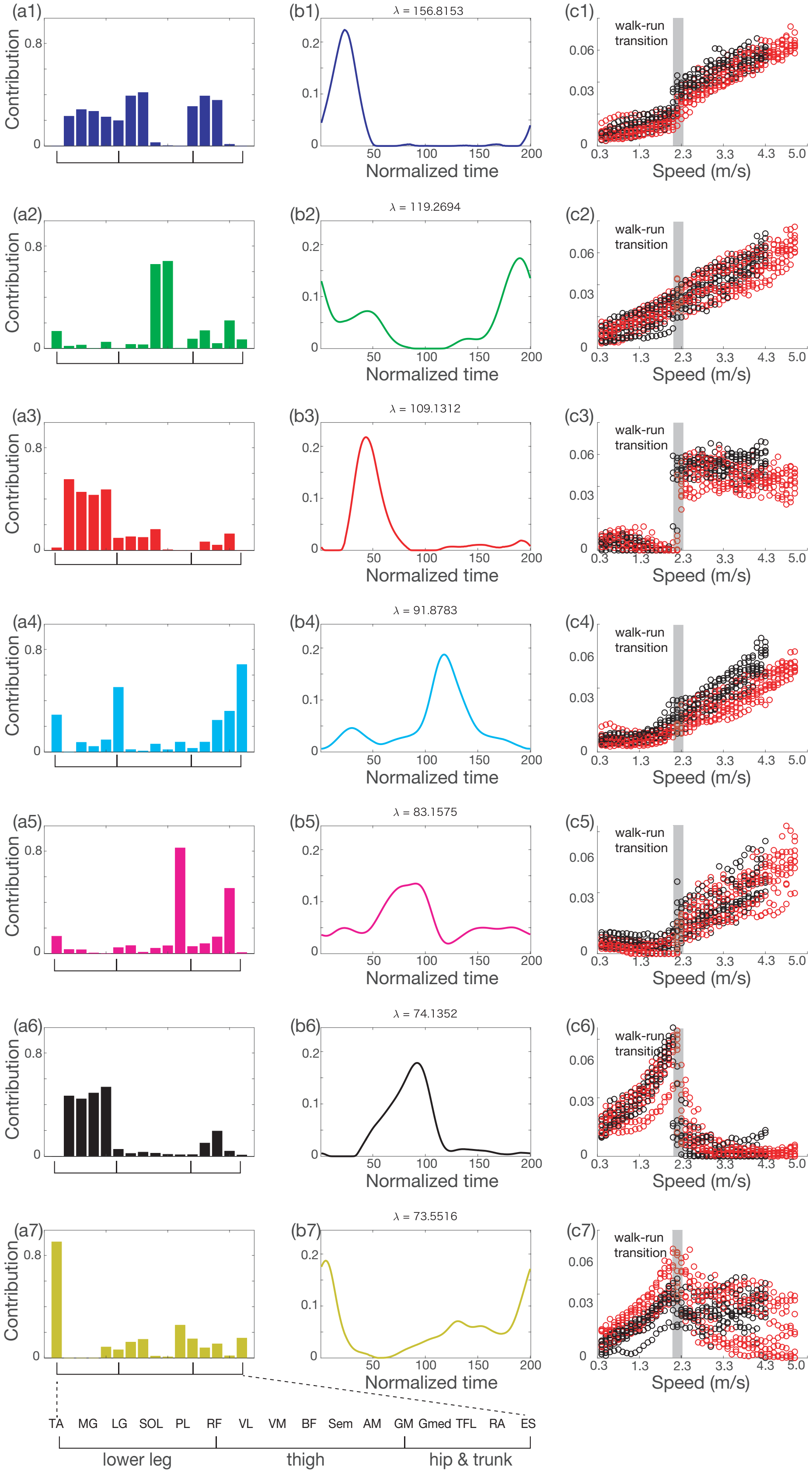

Fig. S2 Tensor decomposition for EMG data. The number of modules was determined based on 75% explained variance.

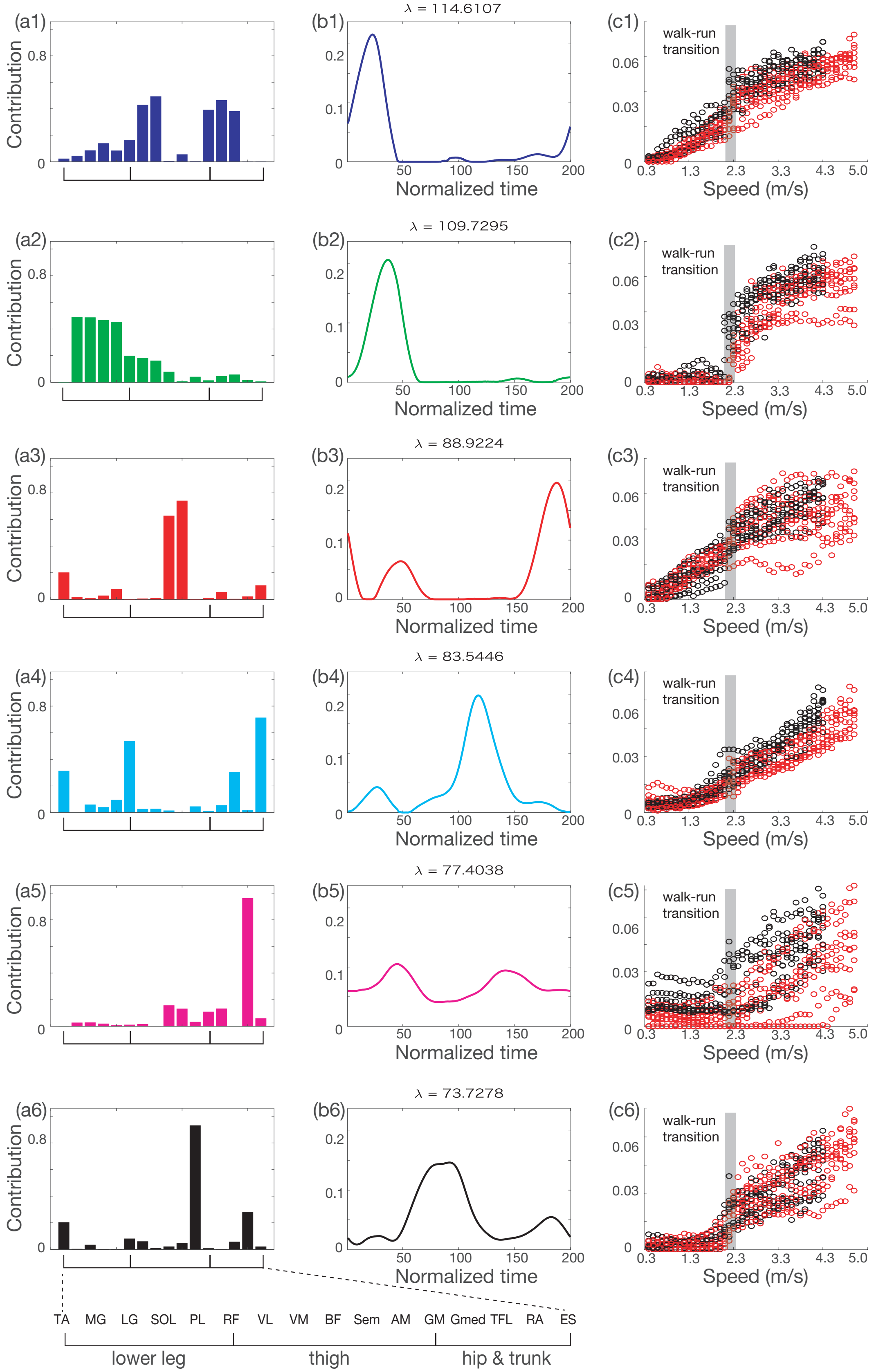

Fig. S3 Tensor decomposition for EMG data. The number of modules was determined based on 80% explained variance. Tensors 1 to 6 are shown.

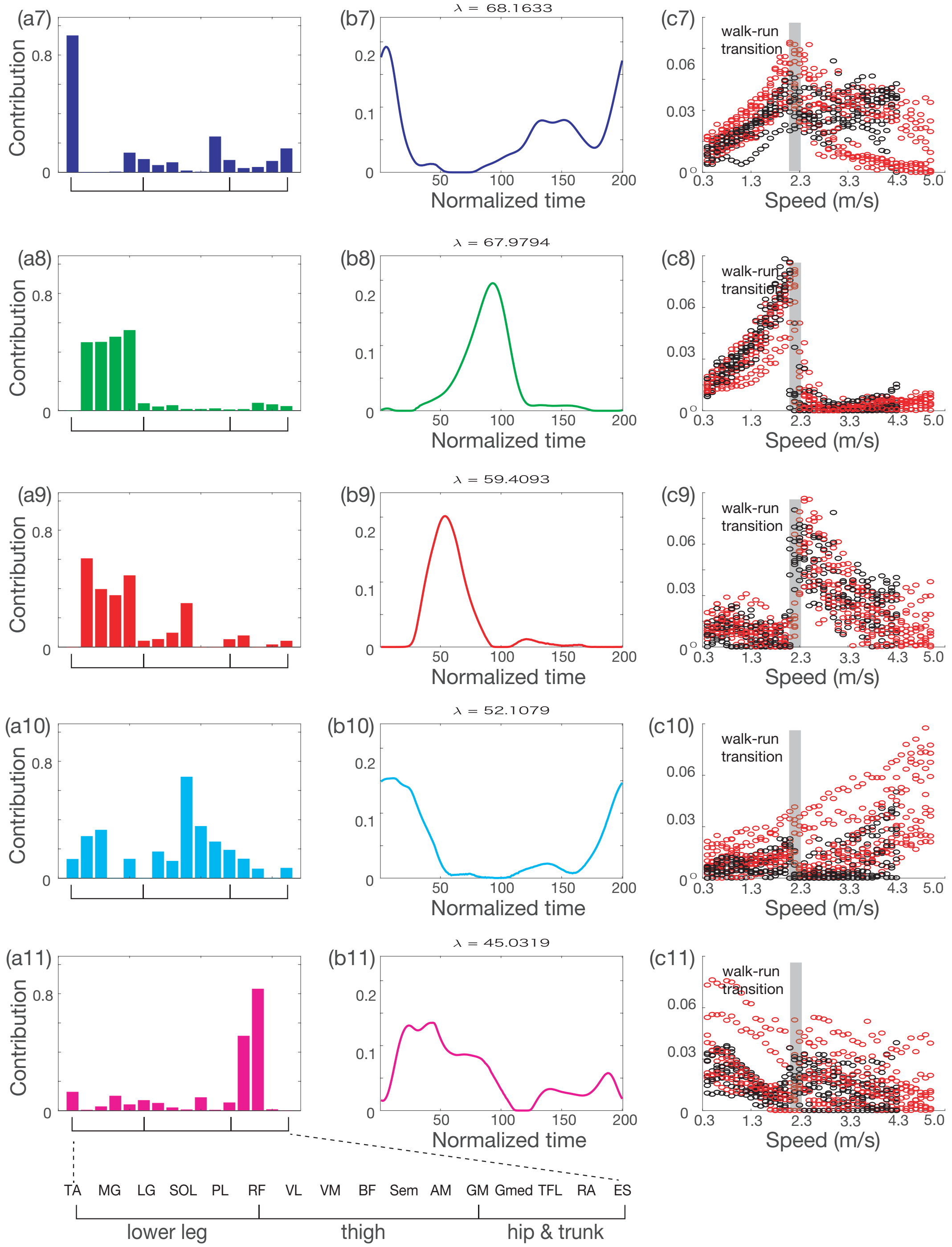

Fig. S4 Tensor decomposition for EMG data. The number of modules was determined based on 80% explained variance. Tensors 7 to 11 are shown.
